# Structural Dynamics of GaN Microcrystals in Evolutionary Selection Selective Area Growth probed by X-ray Microdiffraction

**DOI:** 10.1038/srep04651

**Published:** 2014-04-11

**Authors:** V. Kachkanov, B. Leung, J. Song, Y. Zhang, M.-C. Tsai, G. Yuan, J. Han, K. P. O'Donnell

**Affiliations:** 1 Diamond Light Source Ltd, Diamond House, Chilton, Didcot, Oxfordshire, OX11 0DE, UK; 2 Department of Electrical Engineering, Yale University, 15 Prospect St, New Haven, CT, 06511, USA; 3 Department of Physics, SUPA, University of Strathclyde, Glasgow, Scotland, G4 0NG, UK

## Abstract

A method to grow high quality, single crystalline semiconductor material irrespective of the substrate would allow a cost-effective improvement to functionality and performance of optoelectronic devices. Recently, a novel type of substrate-insensitive growth process called Evolutionary Selection Selective Area Growth (ES-SAG) has been proposed. Here we report the use of X-ray microdiffraction to study the structural properties of GaN microcrystals grown by ES-SAG. Utilizing high resolution in both direct and reciprocal spaces, we have unraveled structural dynamics of GaN microcrystals in growth structures of different dimensions. It has been found that the geometric proportions of the growth constrictions play an important role: 2.6 μm and 4.5 μm wide growth tunnels favor the evolutionary selection mechanism, contrary to the case of 8.6 μm growth tunnels. It was also found that GaN microcrystal ensembles are dominated by slight tensile strain irrespective of growth tunnel shape.

The development of modern electronics industry relies heavily upon processes designed to produce semiconductor materials in the form of thin films. Epitaxial growth has been a mainstay of the semiconductor industry, enabling fabrication of electronic devices for a wide range of applications^1^. However, a fundamental requirement of epitaxial growth is the availability of crystalline substrates lattice-matched to the material of the film; when this is not realized, lattice mismatch becomes a major obstacle, deteriorating the crystalline quality of epitaxial layers through the introduction of strain and defects^2^,^3^. To circumvent the lattice-matching requirement it was suggested to use artificially patterned amorphous or polycrystalline substrates for deposition^4^,^5^. The ability to grow single crystalline semiconductor material on an amorphous surface would allow a selection of functional substrates to complement or improve the functionality of devices. For example, the use of transparent and/or flexible substrates for GaN-based Light Emitting Diodes (LEDs), or thermally and electrically conductive metal foil substrates for electrical devices, can be realised. Recently, some of the present authors reported a novel type of substrate-insensitive growth process named as Evolutionary Selection Selective Area Growth (ES-SAG)^6^. The ES-SAG process is based on a mechanism defined as evolutionary selection^7^ whereby the fastest-growing crystal grains overtake their slower neighbors and become dominant; an engineered growth constriction *filters* misaligned grains thus forming a polycrystalline layer into a nearly monocrystalline one. The process, depicted in Fig. 1, utilizes lithographic techniques and selective area growth. A typical growth sequence consists of two stages, and within each stage the ES principle will reduce the degrees of freedom in orientation. The first stage is the deposition of an AlN film, with a preferred orientation set by the ES principle in the direction perpendicular to the substrate surface. Then, a SiO_2_ dielectric structure is fabricated to confine growth in two dimensions. Subsequent growth on the previously defined textured seed, through the confined structure, will allow the number of grains of the growing material, originally nucleated on the seed, to be reduced in a direction parallel to the substrate surface. However, to understand the ES-SAG process fully requires a spatially resolving characterization technique that can assess both strain and structural quality of the material of interest on the microscale.

X-ray diffraction is a very sensitive and non-invasive structural tool. Recent advances in X-ray optics have lead to the development of various micro- and nanofocusing optical elements^8^. The use of focused X-ray beams brings spatial resolution to diffraction analysis, thereby increasing the functionality of the technique^9^,^10^. In this work we report the investigation of growth dynamics of GaN microcrystals in ES-SAG using high-resolution X-ray microdiffraction in three dimensions.

## Results

Fig. 2 shows representative Scanning Electron Microscopy (SEM) images of the sample studied. As the proposed idea assumes that numerous crystallites nucleate on the AlN seed, the GaN/AlN interface is shown magnified in Figs. 2 (d),(e) and (f). It is seen that voids in the material form lines which propagate from the AlN seed along the growth direction. This is a first indication that GaN microcrystals form during growth, with the voids defining their boundaries. Further along the growth direction, for the narrower channels, the voids disappear, while for the wider channels, they can propagate through the entire length of the tunnel. Characterization by Electron Backscatter Diffraction (EBSD) indicates that lateral growth through the tunnel structure enables an evolutionary selection process to take place, resulting in a large single-crystal GaN on the completely covered SiO_2_ substrate surface^6^. EBSD also shows that initially nucleated GaN grains are randomly oriented in-plane but follow the (0001) texture of the AlN seed. However, the angular resolution of the EBSD setup was no better than ~1 degree; thus a technique with higher angular resolution is required for detailed characterization of quality and strain state of GaN microcrystals.

The concept of the X-ray microdiffraction experiment is depicted in Fig. 3. The focused X-ray beam probed structural properties of GaN microcrystals in the growth tunnels. The angle of incidence *ω* was scanned around the (0002) reflection of GaN with an area detector positioned at the expected *2θ* angle. The footprint of the focused X-ray beam on the sample was ~3.4 × 14.0 μm FWHM for the (0002) reflection. The sample was oriented in such a way that the longest dimension of the X-ray beam was perpendicular to the growth direction. Maxima of intensity are observed when the Bragg condition is satisfied for individual microcrystals. On the area detector the position of a diffraction peak can be used to calculate the absolute modulus of the scattering vector Q; the width of a diffraction peak on the area detector is equivalent to a rocking curve scan, i.e. it is indicative of crystalline quality. The experiment resembles a powder diffraction experiment with (0002) reflections from *individual* grains arranged in a partial Debye-Scherrer ring; partial since there is a preferential orientation set by the AlN growth “seed”. The accuracy of the experiment is estimated to be ±0.0003 Å^−1^ along the [0002] direction in reciprocal space. Note that each microcrystal can be identified by its unique coordinates in the 3D angular space: viz. the incidence angle and two position coordinates on the area detector. The focused X-ray spot *enhances* microcrystal identification by limiting the number probed at any one time. The divergence of the incident beam does not degrade the resolution in reciprocal space since each microcrystal acts as a tiny analyzer crystal thereby reducing the angular spread of the diffracted X-ray beam on the area detector. No correlation was found between the intensities and the broadening of X-ray diffraction peaks, suggesting that due to short integration times (1–2 sec) the measurements were not sensitive to grains small enough for the Scherrer equation to play a substantial role.

Fig. 4 shows the statistical distribution of intensity, the crystalline quality (indicated by the FWHM distribution of diffraction peaks) and the modulus of the scattering vector, Q(0002), for microcrystals grown in tunnels of three different widths. Four growth tunnels of each width were probed along the growth direction. For the statistical analysis for each probed growth tunnel a single *ω* scan with the highest intensity was selected to avoid double counting of microcrystals. Figs. 4(a), (d), (g) show microcrystal intensity distributions for tunnels of different width. The intensity of the X-ray diffraction peaks is proportional to the volume of the microcrystals and it is used here as a guide to their size. A typical intensity distribution has a tail of high intensity peaks corresponding to relatively large microcrystals; average intensity is 268 counts/sec, 159 counts/sec and 92 counts/sec for 8.6 μm, 4.5 μm and 2.6 μm wide tunnels, respectively. Therefore on average GaN microcrystals are biggest in the 8.6 μm wide tunnels and smallest in the 2.6 μm wide tunnels as we might expect. Figs. 4(b), (e), (h) show the distribution of X-ray diffraction peak FWHMs for microcrystals in growth tunnels of different width. Generally, FWHM distributions are broad with a lower limit defined by the detector resolution of 23 arcsec and an upper limit reaching 130 arcsec. Average FWHM is 52.0 arcsec, 51.9 arcsec and 50.8 arcsec for 8.6 μm, 4.5 μm and 2.6 μm wide tunnels, respectively. For reference, these values of crystalline quality are about a tenth of the FWHM reported for GaN on Si substrates^11^, a third of that typical of HVPE grown GaN^12^ and three times larger than that of the best bulk GaN substrates^13^.

Figs. 4(c), (f), (i) show the distributions of Q(0002). The average values of Q(0002) are 2.4261 Å^−1^, 2.4253 Å^−1^ and 2.4255 Å^−1^ which correspond to tensile strains of 0.10%, 0.07% and 0.08% for microcrystals in 8.6 μm, 4.5 μm and 2.6 μm tunnels, respectively. In unstrained GaN the *c* lattice constant value is 5.1850 Å^14^ which corresponds to a Q(0002) of 2.4236 Å^−1^; any deviation from this value indicates the presence of strain in a microcrystal. It is immediately obvious that, for the majority of microcrystals, the Q(0002) values are higher than what is expected for strain-free GaN. Assuming that elasticity theory is valid, it is an indication of the tensile strain dominating microcrystal ensembles irrespective of the growth tunnel geometry. In other words, *c* lattice parameter is compressed on average while *a* lattice parameters are stretched. However, it is not possible to deduce the direction of the biaxial strain from a symmetric reflection.

Fig. 5 compares X-ray diffraction measured along the growth direction for tunnels of different geometry. Utilizing the spatial resolution provided by a microfocused X-ray beam, two regions in the growth tunnels were probed: one close to the GaN/AlN interface and another 4 μm further along the growth direction. This separation is close to three times standard deviation of the beam size and thus there is no overlap between the two probed volumes. The structural comparison of these two regions reveals the dynamics of the growth process for GaN microcrystals in ES-SAG. Figs. 5(a), (d), (g) show the dependence of maximal intensity registered by the detector as a function of incidence angle ω. Figs. 5(b), (e), (h) and Figs. 5(c), (f), (i) compare the dynamics of crystalline quality and strain state of GaN microcrystals for tunnels of different width. It is immediately noticeable that the growth dynamics in 8.6 μm growth tunnel is different from that in 4.5 μm and 2.6 μm tunnels. In 8.6 μm tunnels, there are more GaN microcrystals further away from the interface and they are bigger *at the same time*. The average FWHM actually increases from 38.5 arcsec to 57.1 arcsec and the average tensile strain increases from 0.05% to 0.09%. The structural dynamics changes completely in narrower growth tunnels. GaN microcrystals become bigger as their number *decreases*. The average crystalline quality improves from 64.2 arcsec to 56.7 arcsec and from 65.1 arcsec to 45.1 arcsec for 4.5 and 2.6 μm tunnels respectively. Regarding the average strain state, the tensile strain relaxes from 0.09% to 0.07% and from 0.12% to 0.06% for 4.5 and 2.6 μm tunnels respectively. The trend of tensile strain relaxation and improvement of crystalline quality is more pronounced for the narrowest 2.6 μm growth tunnel. Simultaneous increase of the size and number of microcrystals in the case of 8.6 μm tunnels cannot be explained by the peculiar triangular shape of the GaN/AlN interface as shown on Fig. 2(f). For narrower growth tunnels, the volume of probed material is also higher further from the GaN/AlN interface, since roughly half of the X-ray beam falls on AlN when the beam is on the interface. However, a *decrease* in the number of grains is still observed as the volume of material probed *increases*. This is the key indication that evolutionary selection is taking place in 4.5 and 2.6 μm tunnels but not in 8.6 μm tunnels.

Since each microcrystal can be uniquely identified by its location in 3D angular space, it is possible to trace the evolution of an individual microcrystal along the growth direction and such an analysis is shown in Fig. 5 for some of the largest examples as we are interested only in those that survive and grow during ES-SAG. Three large microcrystals can be traced back to the start of growth in the 8.6 μm growth tunnel. Remarkably, the strain state evolution for the three largest microcrystals points to a common initial tensile strain of ~0.06%. Only a single microcrystal can be traced back in both the 4.5 μm and 2.6 μm growth tunnels. Note that the largest microcrystal in the 2.6 μm growth tunnel incorporates no additional strain as it grows.

## Discussion

Evolutionary selection is the key principle of the ES-SAG process and it is based on competition between crystal grains during growth. The dependence of the structural dynamics of GaN microcrystals on the growth tunnel width indicates that the geometric proportions of the growth constrictions play an important role; at the same time pointing to the spatial scale on which evolutionary selection takes place. The growth dynamics in 4.5 μm and 2.6 μm tunnels conforms to the idea of evolutionary selection. The best outcome in terms of crystalline quality and strain state of GaN microcrystals is observed for the narrowest 2.6 μm growth tunnels. On the other hand, the growth dynamics in the widest 8.6 μm growth tunnels does not show selection of microcrystals; instead it is their diversification that is uniquely observed in these high-resolution studies which reveal the growth dynamics of spatially traceable individual microcrystals.

Another interesting observation is that GaN microcrystal ensembles are always grown with slight tensile strain. This tensile strain is a manifestation of residual stress in the microcrystals. It is worth noting that the material system under investigation shares more similarity with polycrystalline thin films than with epitaxial layers. A lot of work has been devoted to investigations of intrinsic residual stress in polycrystalline metallic thin films^15^,^16^,^17^,^18^,^19^. It is generally assumed that the residual stress can be classified into three main components: intrinsic, thermal and external. Formation of grain boundaries during coalescence of grains was first suggested as a mechanism for generation of intrinsic residual stress^15^. Later, a quantitative evaluation of the intrinsic residual stress was proposed based on size-dependent phase transition of nanograins^19^,^20^. In this model it is assumed that the phase transition from liquid to solid induces a volume change due to thermal contraction that leads to intrinsic residual stress. Thermal residual stress is caused by the difference in thermal expansion coefficients between the film and the substrate, whereas the main cause of external residual stress is the oxidation and incorporation of impurities. For GaN on SiO_2_, thermal stress can be ruled out since as it would lead to tensile strain values ~0.5%, higher than the observed values. The extrinsic stress due to diffusion of aluminium atoms and oxidation can also be ruled out as GaN growth is carried out after etching of previously grown AlN and GaN is not susceptible to oxidation under normal conditions. The only viable explanation for tensile strain is the intrinsic stress. Interestingly, for thin metallic films it was shown that intrinsic residual stress is generally tensile and contributes significantly more to the overall stress in comparison to thermal stress^15^,^17^,^18^. We suggest that the observed strain, which dominates the microcrystal ensembles, originates from intrinsic residual stress generated in microcrystals during ES-SAG. Assuming that residual stress is biaxial and isotropic in the basal plane it is possible to estimate residual stress using elastic theory^21^: 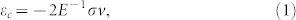
where *ε_c_* is the strain component along the *c*-axis, *σ* is biaxial stress, *E* is Young's modulus and *ν* is Poisson's ratio. Using reported values for Poisson ratio ν ≈ 0.23^21^ and Youngs'modulus *E* = 330 GPa^22^, the tensile strain of 0.10% would correspond to residual stress *σ* ≈ 0.72 GPa. However, the full understanding of intrinsic residual stress in ES-SAG requires detailed modeling similar to that done in Ref. 19, 20. It is the key to the further development and application of this novel growth method.

## Methods

### Evolutionary Selection Selective Area Growth

**Figure 1:**
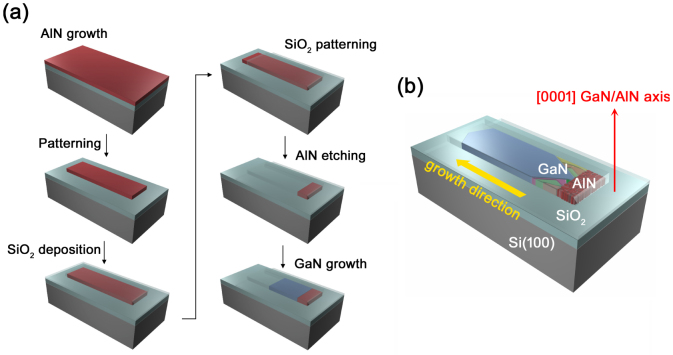
Fabrication and concept of Evolutionary Selection Selective Area Growth. (a) processing steps for fabrication of SiO_2_ growth tunnels; (b) Schematics of the ES process in the growth tunnel: GaN microcrystals retain the (0001) texture of the AlN seed while randomly oriented in-plane.

**Figure 2:**
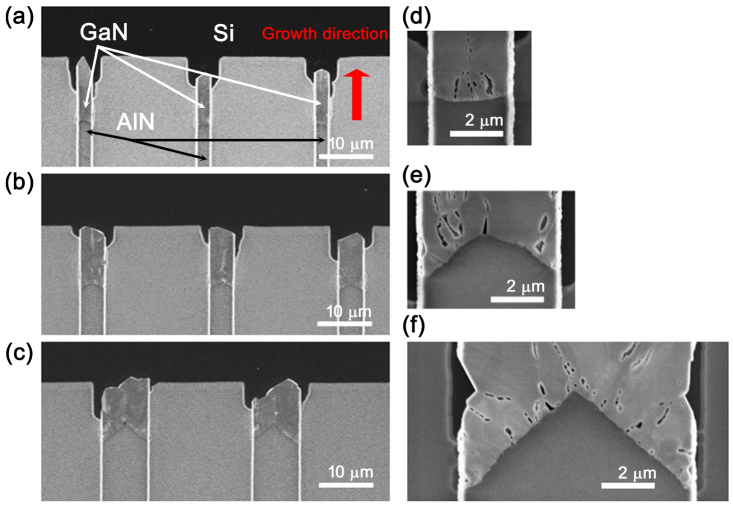
Top view SEM images of GaN crystals grown by ES-SAG. (a) 2.6 μm, (b) 4.5 μm, and (c) 8.6 μm wide growth tunnels. The SiO_2_ tunnel confined mask structure has been removed by buffered oxide etch to expose the GaN and AlN seed, as well as the Si(100) substrate. Scale bar is 10 μm. Magnified view of the GaN/AlN interface is shown in (d), (e) and (f) for representative 2.6 mm, 4.5 mm and 8.6 mm wide grown GaN.

**Figure 3:**
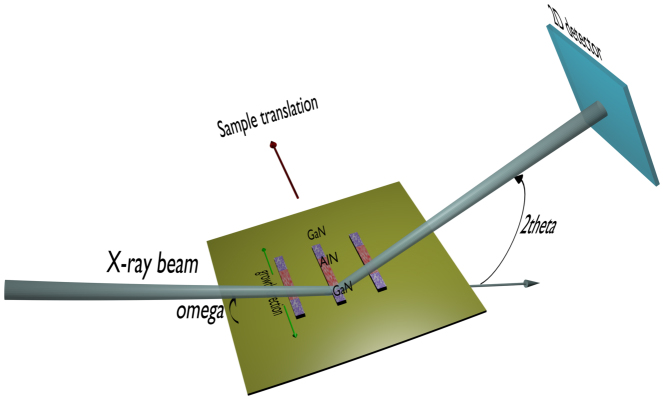
Schematics illustrating microdiffraction experiment. Focused X-ray beam has been used to probe GaN microcrystals in growth tunnels. The sample was mounted on the diffractometer x-y translation stage in such a way that the longest dimension of the X-ray beam was perpendicular to the growth direction.

**Figure 4:**
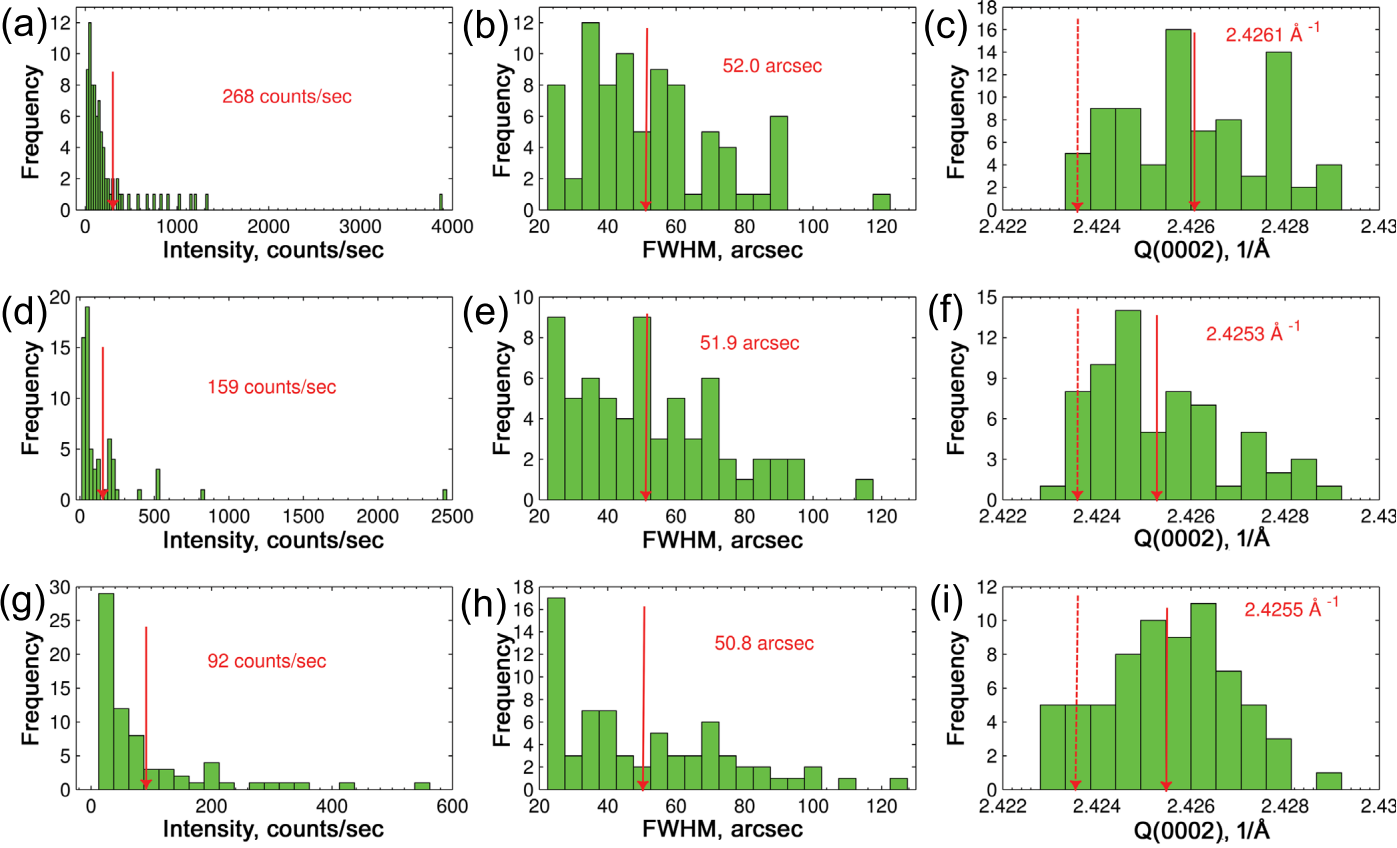
Statistical distibution of intensity, FWHM and modulus of scattering vector Q(0002) for X-ray diffraction peaks of GaN microcrystals. 8.6 μm (a), (b), (c), 4.5 μm (d), (e), (f) and 2.6 μm (g), (h), (i) wide growth tunnels. The average values are indicated by red arrows. In (c), (f), (i) dashed red arrows indicate Q(0002) values for strain-free GaN.

**Figure 5:**
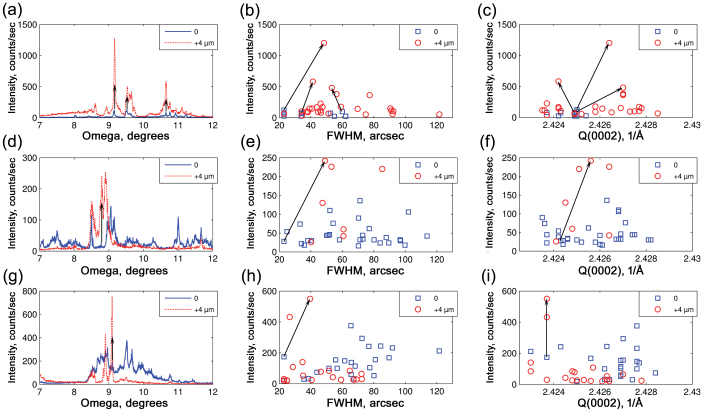
Structural evolution of GaN microcrystals along the growth direction. 8.6 μm (a), (b), (c), 4.5 μm (d), (e), (f) and 2.6 μm (g), (h), (i) wide growth tunnels. The first probed area close to the GaN/AlN interface is compared to a second one which is 4 μm further along the growth direction. In (b), (e), (h) the average FWHM changes from 38.5 to 57.1 arcsec, from 64.2 arcsec to 56.7 arcsec, and from 65.1 arcsec to 45.1 arcsec, respectively. In (c), (f), (i) the average Q(0002) changes from 2.4248 Å^−1^ to 2.4258 Å^−1^, 2.4257 Å^−1^ to 2.4253 Å^−1^, and 2.4265 Å^−1^ to 2.4251 Å^−1^. The black arrows trace the evolution of individual microcrystals. The accuracy of the experiment in reciprocal space is estimated to be ±0.0003 Å^−1^.

GaN growth on SiO_2_ was performed by the ES-SAG method. The processing steps for ES-SAG are shown in Fig. 1. A textured (0001) AlN film, 0.65 μm thick, was deposited by rf-magnetron sputtering on a SiO_2_-covered Si(100) wafer. The AlN is patterned by standard photolithography and reactive-ion etching into 15 μm long stripes with widths of 2.6 μm, 4.5 μm and 8.6 μm. Plasma-enhanced chemical vapor deposition (PECVD) of 800 nm SiO_2_ was then performed to cover the AlN stripes. In a second photolithography step, SiO_2_ was removed on the sides of the AlN patterns by a buffered oxide etch. AlN is then etched back using 25% tetramethylammoniumhydroxide (TMAH) at 65°C. Note that the thickness of the AlN film defines a vertical size for growth tunnels. After substrates were cleaned thoroughly, they were loaded in a metal-organic chemical vapor deposition (MOCVD) system for GaN growth. Trimethylgallium (TMGa) and ammonia (NH_3_) were used as sources for gallium and nitrogen, respectively, and H_2_ as a carrier gas. TMGa is introduced at a temperature of 1030°C, 300 mbar, and 0.5 slm NH_3_. Typical longitudinal growth rates of GaN inside the tunnels are 7–12 μm/h.

The determination of the length scales for growth tunnels was based on the spatial scale where evolutionary selection is taking place. Importantly, the length and the width of the tunnels required to reduce the polycrystallinity scale with the nuclei density, which has been experimentally observed to be ~2–4 × 10^4^ cm^−1^ for GaN on AlN in our MOCVD growth conditions. In order to have an effect in a reasonable growth time, this sets an upper bound of ~10 μm on the width of tunnel that should be used for the ES process. The minimum bound is given by practical considerations, as only conventional photolithography is used, in which structures ~2 μm in size are easily fabricated. An intermediate width is chosen for comparison. The exact widths of the structures deviate slightly from design due to process conditions (over/under exposure) during the photolithography, yielding our current measured widths.

### X-ray microdiffraction in three dimensions

The microdiffraction experiments were carried out on beamline B16 at the Diamond Light Source, UK. The X-ray energy was fixed at 12400 eV (1 Å). The X-ray beam was focused by a Beryllium Compound Refractive Lens (CRL) comprising 63 individual components. The focused X-ray beam size was ~3.4 (horizontal) μm × 2.4 μm (vertical) full width at half maximum (FWHM). The horizontal and vertical incident beam divergences were ~0.8 mrad. The required sample rotations and translations were performed using a high precision 5-circle Huber diffractometer with 0.1 millidegree resolution and a Huber XYZ sample stage with 0.5 μm resolution. A Pilatus 300k detector with pixel size of 0.172 × 0.172 mm was used to record 2D X-ray diffraction patterns.

The addition of an area detector to a 5-circle diffractometer makes the setup effectively a 6-circle diffractometer^23^. In order to calculate the components of the scattering vector in the diffractometer frame of reference the following equations have been used: 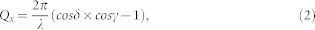

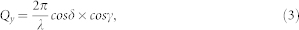

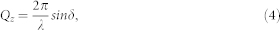
where *δ* is the angle between the detector arm and the horizontal plane, *γ* is the angle between the detector arm and the vertical plane^23^. It is worth noting that *δ* is not the same as the Bragg angle *2θ* which forms the Debye-Scherrer rings on a detector. The angular resolution of the detector, defined by pixel size and detector-sample distance, was ~23 arcsec giving overall angular span of ~3.9° for *γ* and ~3.1° for *δ*.

In the microdiffraction experiment, the incidence angle *ω* is scanned while area detector is fixed at expected *2θ* angle. When the incidence angle matches the Bragg conditions for a microcrystal, an X-ray diffraction spot is observed on 2D detector producing a peak in incidence angle *ω* versus maximal intensity plot i.e *ω*-scan. For clarification, maximal intensity is maximal intensity in a single pixel and not the intensity integrated across all the pixels of the area detector. A broadening of an X-ray diffraction spot along *2θ* (i.e. across the Debye-Scherrer ring) on the 2D detector indicates a spread of lattice parameter. The broadening perpendicular to *2θ*, i.e. tangential to the Debye-Scherrer ring, also known as *χ*-spread, is in this case a complex function of strain and orientation of unit cells *inside a single grain* i.e. crystal deformation.

To extract statistics on GaN grains in growth tunnels, peak positions in *ω*-scans were extracted using a local maxima finding algorithm which utilizes first derivative test and Savitzky-Golay filtering for noise rejection. Thus a 2D image corresponding to a peak in *ω*-scan was extracted and analyzed. The 2D image analysis was done in the following way:

1. Each pixel position in the image was converted into diffractometer angles *δ* and *γ* using simple geometric relations.
2. Angular coordinates of the X-ray diffraction peak were used to calculate the absolute modulus of the total scattering vector Q using relations (2)–(4).
3. The line broadening indicated by FWHM was extracted by fitting each X-ray diffraction peak with a Lorentzian along the *2θ* direction using standard non-linear least squares fit routine.

The MATLAB computing environment was used to carry out data analysis.

## Author Contributions

V.K. carried out the microdiffraction experiment and data analysis. B.L., J.S., Y.Z., M.-C.T., G.Y. and J.H. grew the sample. V.K. wrote the paper with suggestions from K.P.O'D. and B.L.
